# *In silico* Prediction and Validations of Domains Involved in *Gossypium hirsutum* SnRK1 Protein Interaction With Cotton Leaf Curl Multan Betasatellite Encoded βC1

**DOI:** 10.3389/fpls.2019.00656

**Published:** 2019-05-28

**Authors:** Hira Kamal, Fayyaz-ul-Amir Afsar Minhas, Muhammad Farooq, Diwaker Tripathi, Muhammad Hamza, Roma Mustafa, Muhammad Zuhaib Khan, Shahid Mansoor, Hanu R. Pappu, Imran Amin

**Affiliations:** ^1^National Institute for Biotechnology and Genetic Engineering, Faisalabad, Pakistan; ^2^Pakistan Institute of Engineering and Applied Sciences, Islamabad, Pakistan; ^3^Department of Plant Pathology, Washington State University, Pullman, WA, United States; ^4^Department of Biology, University of Washington, Seattle, WA, United States

**Keywords:** cotton leaf curl disease, cotton leaf curl Multan betasatellite, sucrose-non-fermenting 1 kinase, yeast two hybrid, bimolecular fluorescence complementation, pull down assay, begomovirus, geminivirus

## Abstract

Cotton leaf curl disease (CLCuD) caused by viruses of genus *Begomovirus* is a major constraint to cotton (*Gossypium hirsutum*) production in many cotton-growing regions of the world. Symptoms of the disease are caused by Cotton leaf curl Multan betasatellite (CLCuMB) that encodes a pathogenicity determinant protein, βC1. Here, we report the identification of interacting regions in βC1 protein by using computational approaches including sequence recognition, and binding site and interface prediction methods. We show the domain-level interactions based on the structural analysis of *G. hirsutum* SnRK1 protein and its domains with CLCuMB-βC1. To verify and validate the *in silico* predictions, three different experimental approaches, yeast two hybrid, bimolecular fluorescence complementation and pull down assay were used. Our results showed that ubiquitin-associated domain (UBA) and autoinhibitory sequence (AIS) domains of *G. hirsutum*-encoded SnRK1 are involved in CLCuMB-βC1 interaction. This is the first comprehensive investigation that combined *in silico* interaction prediction followed by experimental validation of interaction between CLCuMB-βC1 and a host protein. We demonstrated that data from computational biology could provide binding site information between CLCuD-associated viruses/satellites and new hosts that lack known binding site information for protein–protein interaction studies. Implications of these findings are discussed.

## Introduction

Plant viruses cause considerable damage to quality and crop yield and threaten food security in several parts of the world (Oerke and Dehne, 2004). One of the largest groups of plant viruses is geminiviruses. Family *Geminivirdae* is classified into nine genera, having single-stranded (ss) circular genome encapsidated in a twin icosahedral particle that range in size from 18 to 30 nm (Hanley-Bowdoin et al., 2000). Geminiviruses interact with several proteins in the host to cause changes in their transcription and translation machinery for virus multiplication. Family *Geminivirdae* is divided into nine genera based on their genome organization, insect vectors and host range (Martin et al., 2011). Among them, *Begomovirus* is the largest and most economically important genus, and viruses in this genus cause serious diseases in agronomic and horticultural crops such as cotton, cassava, maize, and tomato (Brown et al., 2015). Besides environmental adaptation, begomoviruses rapidly modify their genetic information to make favorable protein complex in a host to develop tolerance against plants immune system (Brown and Bird, 1992). Typically, begomoviruses are divided into two classes, i.e., monopartite (having a single genomic component), and bipartite (having two genomic components). Interestingly, the Old world (OW) monopartite begomoviruses are often associated with satellites referred to as alphasatellite and betasatellite. Betasatellite encodes a protein, βC1, which is essential for infection. Viruses causing cotton leaf curl disease (CLCuD) are betasatellite-requiring monopartite begomoviruses that cause serious economic damage to cotton (*Gossypium hirsutum* L.) in the Indian subcontinent and Africa (Nawaz-ul-Rehman et al., 2009; Tiendrébéogo et al., 2010).

**FIGURE 1:**
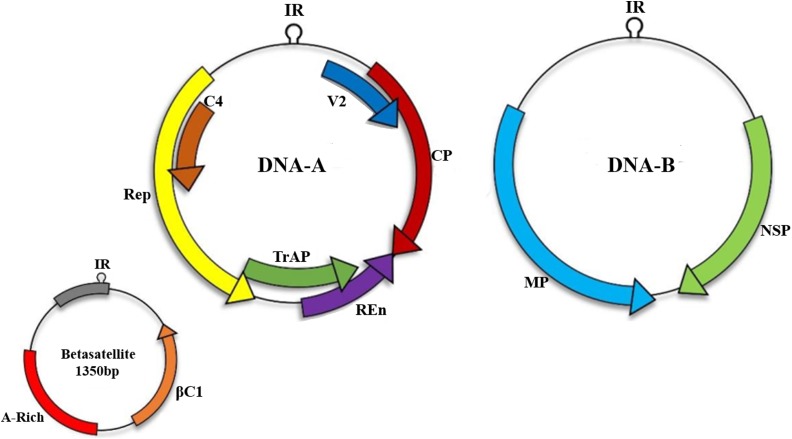
Begomoviruses are transmitted by an insect vector *B. tabaci*. A monopartite begomoviruses requires only DNA-A with associated satellites such as betasatellite to cause infection in host cell while bipartite viruses encodes both DNA-A and DNA-B. Monopartite virus can cause infection with the help of small satellite molecules.

Betasatellites (genus *Betasatellite*, family *Tolecusatellitidae*) are circular ssDNA molecules mostly associated with monopartite begomoviruses of OW (Briddon and Markham, 2001; Fauquet and Stanley, 2003; Zhou et al., 2003; Mansoor et al., 2008; Briddon et al., 2018). Betasatellite was first identified from *Ageratum yellow vein virus* (AYVV)-infected *Ageratum conyzoides* plant (Saunders et al., 2000). The betasatellite DNA is approximately 1350 nucleotides (Briddon et al., 2001, 2008) shown in Figure 1, and is involved in counteracting host transcriptional gene silencing (TGS) and post-transcriptional gene silencing mechanism (PTGS) (Li and Ding, 2006; Hayward et al., 2009). For inducing enhanced pathogenicity, βC1 also augments accumulation of high levels of the helper begomoviruses (Saeed et al., 2007). In addition, it also regulates microRNA levels involved in the host developmental processes (Amin et al., 2011) and interacts with several virus and host proteins (Cheng et al., 2011). Role of this virus protein has been identified in begomoviruses such as βC1, associated with *Tomato yellow leaf curl China virus* (TYLCCNV) infection, interacts with Asymmetric leaves1 (AS1) to prevent normal leaf development and usurp cellular resources by interfering with jasmonic acid (JA) responsive genes to induce infestation by insect vector *Bemisia tabaci* (Yang et al., 2008). Another protein, ubiquitin-conjugating enzyme E3 (SlUBC3), encoded by *Solanum lycopersicum* shows interaction with CLCuMB suggesting that βC1 also interferes with UBC in ubiquitin proteasome pathway (Eini et al., 2009).

Interaction study at domain level was performed for Sucrose-non-fermenting 1 (SNF1)-related kinase (SnRK1) protein present in *S. lycopersicum*. This *S. lycopersicum* encoded SnRK1 protein plays a significant role in phosphorylating Tomato yellow leaf curl China betasatellite (TYLCCNB)-βC1, thus acts as an antiviral protein (Shen et al., 2011). Therefore, sequence and structure based methods at domain level could identify the interaction between CLCuD-causing viruses and host proteins. A recent study revealed that SnRK1 phosphorylates geminivirus encoded Rep protein of *Tomato golden mosaic virus* (TGMV) and mutagenesis study determined the function of interacting domains involved in binding with the virus (Shen et al., 2018). All of these studies indicated that SnRK1 protein is involved in various physiological processes in plants including regulation of energy metabolism and stress signaling during biotic and abiotic stresses (Hulsmans et al., 2016; Wurzinger et al., 2018).

Leading to protein–protein interaction (PPI), high-throughput technologies and bioinformatics data possess information for number of proteins at host side that are monitored during CLCuD development. Geminivirus proteins interact with a large number of host proteins during infection and *in silico* study is a great source to identify putative binding site between host and begomovirus to control CLCuD in future (Malik et al., 2016). So far protein interaction prediction methods have been proposed based on sequence or structure information. However, only sequence or structure based methods do not produce optimal result for inter-species interaction (Zhou et al., 2013). Interaction prediction strategy with combination of sequence and structure based methods showed higher sensitivity in identifying the interface region(s) between virus and its host (Hamp and Rost, 2015).

Here, we investigated cotton leaf curl Multan betasatellite (CLCuMB)-encoded βC1 protein’s binding with *G. hirsutum*-encoded SnRK1α (GhSnRK1) protein at domain level. By using the sequence and structure information about the CLCuMB-βC1 and GhSnRK1 complex, it was determined that the α-helix in CLCuMB-βC1 where GhSnRK1 possessing ubiquitin-associated (UBA) and autoinhibitory sequence (AIS) domains are responsible for interaction during CLCuD. This *in silico* interaction data was verified by three independent experimental methods, yeast two hybrid (Y2H), bimolecular fluorescence complementation (BiFC) and pull-down assays. Findings provided a deeper understanding and insights into interactions underlying the begomovirus-host protein interactions.

## Materials and Methods

### *In silico* Tools for Interaction and Binding Site Prediction

Multiple approaches were employed to identify interaction between virus CLCuMB and host GhSnRK1 protein. Host domain information was deduced from NCBI conserved domain database (Marchler-Bauer et al., 2016), InterPro at EMBL-EBI (Guo et al., 2008), PROSITE (Sigrist et al., 2012), and ThreaDom (Xue et al., 2013). After domain localization, three-dimensional structure of GhSnRK1, its domains and CLCuMB-βC1 were also predicted using I-TASSER (Zhang, 2008). Sequence alignment was done using local and global protein alignment tools at EMBOSS (Rice et al., 2000) and root mean square deviation-RMSD was observed in PyMOL with structure alignment (DeLano, 2009). To identify interaction in terms of binding affinity ΔΔG (change in Gibbs free energy), sequence based method PPA-Pred (Yugandhar and Gromiha, 2014) and structure-based method PRISM (Baspinar et al., 2014) and PRODIGY (Xue et al., 2016) were used. Further, to determine binding site, binding site prediction methods including PSIVER (Protein–protein interaction SItes prediction serVER) (Murakami and Mizuguchi, 2010), Bspred (Mukherjee and Zhang, 2011), NSP-HomPPI (Non-partner-specific HomPPI) (Xue et al., 2011), and PredictProtein (Yachdav et al., 2014) were employed. These sequence-based methods depend on threshold default parameters for identification of binding site such as PSIVER generated residue based binding site using two threshold values >=0.37 (optimum) and >=0.56 (higher specificity). Bspred scoring is also based on neural network (NN) showing NN score >-0.1 as an optimum value. NSP-HomPPI identified very few residues in safe-mode zone (optimum score). Relative accessible surface area based structure prediction methods VORFFIP (Segura et al., 2011), PSIVER (Mukherjee and Zhang, 2011), ProMate (Neuvirth et al., 2007), and PredUS (Zhang et al., 2011) were used to identifying possible binding sites. Domain-based interaction was confirmed with machine learning methods PPiPP (Ahmad and Mizuguchi, 2011), PRISM (Baspinar et al., 2014), and PAIRPred (Minhas et al., 2014) which was further confirmed with ZDOCK (Pierce et al., 2014) and Docking2 at ROSETTA v3.2 (Lyskov et al., 2013). List of all sequence and structure-based methods are mentioned in Supplementary Figure S1. ZDOCK gives a blocking option to exclude residues (block contact) to filter output result while performing protein-protein docking. With this option, UBA and AIS domain were blocked and only kinase domain (KD) and C-terminal domain (CTD) were allowed for binding. Another job processed with UBA and AIS domain without KD and CTD domain to determine interaction at domain level. Computational docking methods retrieved the detailed information about surface residues of SnRK1 and βC1 in tomato and cotton using their 3D structures.

### Plant Lines and Growth Conditions

For *in planta* protein interaction study, wild type *Nicotiana benthamiana* seeds were grown in Sunshine Mix LC1 (Sun Gro Horticulture) in growth chambers with 120 μmol photons m^-2^ s^-2^, 16 h light/8 h dark cycle, 20°C. A distinct isolate of cotton leaf curl Multan betasatellite (acc AM774307) was used as inoculum source for this study. SNF1-related kinase protein GhSnRK1 from cotton (*G. hirsutum*) cultivar UA222 resistant variety has been used for isolation of host proteins. All the GhSnRK1 domains based data were also generated from the same cultivar UA222.

### RNA Isolation and Genes Amplification

Total RNA was extracted using RNeasy Plant Mini Kit (Qiagen) from virus infected sample and cotton plant following the manufacturer’s instructions. Purified RNA was then reverse transcribed to generate cDNA using a RevertAid first strand cDNA synthesis kit (Thermo Fisher Scientific). RT-PCR based amplified products were then inserted into pENTR-D-TOPO vector (Invitrogen). Virus and host sequences are available at GenBank (CLCuMB, AM774307; GhSnRK1, MH626512). Further, Gateway cloning based amplicons were used for all destination vectors using specific primers (Supplementary Table S1).

### Yeast Two Hybrid Assay

The full length host gene GhSnRK1, its four domains and viral gene CLCuMB were sub-cloned into yeast-2-hybrid plasmids using gateway LR clonase enzyme (Life Technologies). For destination vectors, pEZY202 and pEZY45 (Addgene plasmid # 18704 and 18705) (Guo et al., 2008) plasmids were used, possessing LexA DNA-binding domain (DBD) and B42 activation domain (AD), respectively. Yeast (*Saccharomyces cerevisiae*) strain EGY48 possessing pSH18-34 was used in lithium acetate yeast transformation procedure described as (Gyuris et al., 1993). GhSnRK1 and its four domains were cloned separately into bait vector pEZY202. CLCuMB-βC1 was cloned into prey vector, pEZY4,5 using Gateway cloning. Successful transformation was observed on minimal SD Base/Gal/Raf with double dropout supplement (DDO) -His/-Ura medium (Clontech). Yeast cell lines yielding bait plasmids were then cotransformed with B42-βC1 as a prey to produce diploid cells. Pre-screening of positive clones were observed on the minimal SD Base/Gal/Raf with triple dropout medium -His/-Trp/-Ura (TDO/+L) and Quadruple dropout medium -His/-Leu/-Trp/-Ura (QDO/-L). The autoactivation step was preformed using three different constructs having GhSnRK1^bait^/empty^prey^, empty^bait^/βC1^prey^ and empty^bait^/empty^prey^, respectively. *Tomato spotted wilt virus*-encoded nucleoprotein (N) protein (TSWV-N) was used as a positive control to ensure the performance of the transformation protocol and screening steps during this assay (Tripathi et al., 2015). To further assess the molecular strength of the GhSnRK1 with CLCuMB-βC1, diploid cells were grown and spotted on agar plates containing SD-His/-Leu/-Trp/-Ura supplemented with 3-Amino-1,2,4-triazole (3-AT) ranging in a serial dilution of 0, 10, 20, and 30 mM.

### BiFC Assay and Fluorescent Protein Expression Analysis

For *in vivo* virus host interaction study, binary vectors based on yellow fluorescent marker (pSITE-nEYFP-C1 and pSITE-cEYFP-C1) were obtained from Arabidopsis Biological Research center (ABRC; Ohio). Host proteins were cloned at “N” terminal and CLCuMB-βC1 at “C” terminal. All clones were confirmed using gene specific primers (Supplementary Table S1). For *Agrobacterium* transformation, all the BiFC constructs were electroporated using GV3101 strain and agroinoculation was performed at OD_600_ value of 0.6–1.0 by infiltrating each BiFC construct to 3–6 *N. benthamiana* leaves, fused with CFP fluorescent marker targeted to histone 2B. These agro infiltrated plants were grown under constant light at 25°C. After 26–48 h incubation, confocal microscopy was performed by keeping leaf tissues on wet mounted slides. Fluorescence for virus host pair was detected using CFP Ex-458 nm/Em-480 nm, YFP laser Ex-514 nm/Em-527 nm, and CFP/YFP FRET Ex-458 nm/Em-527 nm laser. Images were acquired using Leica TCS SP8 X microscopy at 20× dry, 40× dry, and 63× oil for fine detail images and LAS X software were used to analyze the fluorescence signals.

### Pull Down Assay

For *in vitro* study, Maltose binding protein (MBP) pull down assay was performed as described in detail previously (Hapiak et al., 2008). MBP-tagged “bait” crude proteins pMAL-c2X-GhSnRK1/, domains-KD/UBA/AIS/CTD, and GST-tagged “prey” crude protein pDEST15-βC1 were purified from *E. coli* BL21 (DE3) strain using sonication method and mixed together to produce three tubes as “Load,” “Flow-Through,” and “Elution” after consecutive washes and final elution step. All purified proteins with their controls (MBP alone with empty expression vector or transformed with GhSnRK1 or CLCuMB-βC1) were separated on sodium dodecyl sulfate-polyacrylamide gel electrophoresis (SDS-PAGE) followed by Western blotting using monoclonal anti-GST antibody (primary) which was further probed with secondary antibody goat HRP-conjugated anti-rabbit IgG (Bio-Rad). Positive signals were acquired on short exposure x-ray films using the ECL method based on Versa Doc imaging system (Bio-Rad) following the manufacturer’s details to determine the interacting domains of host with CLCuMB-βC1.

## Results

### *In silico* Prediction of Host and Viral Protein Structures

**FIGURE 2:**
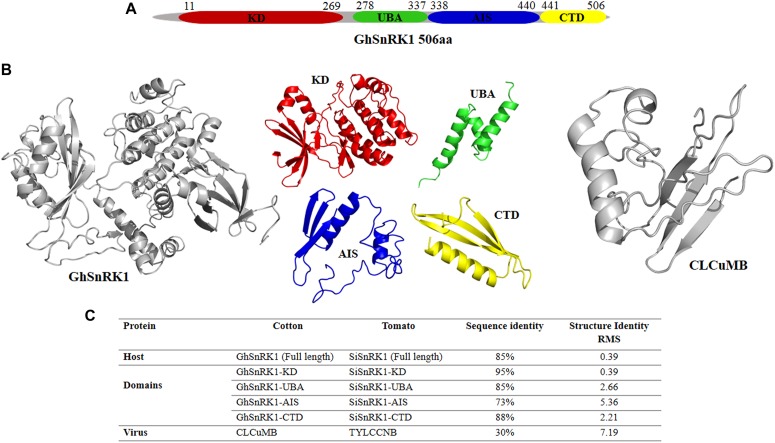
Domain identification and structure prediction for interaction study. **(A)** Full length GhSnRK1, and its domains are predicted using several databases based on sequence information. GhSnRK1 protein and its domains are individually analyzed with CLCuMB-βC1 to identify interaction within a domain using bioinformatics approach. **(B)** PDB structures are determined based on C-score using I-TASSER. Full length SnRK1 protein (gray) and its domains are shown here with different colors. Virus protein structure (gray) is shown on the right side. **(C)** Sequence and structure analysis using alignment tools shows AIS domain among all four domains possess low similarity between tomato coded SnRK1 protein and cotton coded SnRK1 protein.

GhSnRK1 and its domain-based information was retrieved using online data servers shown in the Section “Materials and Methods.” This sequence analysis showed GhSnRK1 encodes a 506 aa protein, consisting of four domains, kinase domain KD (259 aa), Ubiquitin-associated domain UBA (59 aa) and autoinhibitory sequence AIS (104 aa), and C-terminal domain CTD (65 aa) (Figure 2A). Structures for full length host GhSnRK1 protein and its domains were predicted using I-TASSER (Zhang, 2008) as tertiary structures of these proteins are not available in PDB. Similarly, the structure of CLCuMB-βC1 protein was also predicted using I-TASSER (Zhang, 2008). Among all models predicted by I-TASSER, the most accurate model was selected based on high C-score (Figure 2B). *C*-score is a confidence score used to estimate the accuracy of the predicted models in the range of -5 to +2. *G. hirsutum*-coded SnRK1 protein structure and CLCuMB-βC1 were used for domain-based interaction prediction, whereas *Solanum lycopersicum*-coded SnRK1 and TYLCCNB-βC1 complex were used as a control.

### Sequence and Structure Alignment

Sequence and structure alignment of TYLCCNB with CLCuMB and host protein SiSnRK1 with GhSnRK1 using Water (Rice et al., 2000) and PyMOL (DeLano, 2009) showed that the host proteins are 85% identical in sequence with a root mean squared deviation (RMSD) of only 0.39 Å in their predicted structures. At domain level, these host proteins have low similarity between AIS domains of the two proteins (73% sequence identity) (Figure 2C). However, βC1 proteins from TYLCCNB and CLCuMB possess only 30% sequence identity with an RMSD of 7.19 Å (Figure 2C). This low sequence to structure identity between CLCuMB and TYLCCNB shows that it is impossible to deduce a possible interaction of CLCuMB-βC1 with SnRK1 based on the known interaction between TYLCCNB-βC1 with SnRK1.

### Binding Affinity Prediction

To determine the possible interaction between CLCuMB-βC1 and GhSnRK1, we used several binding affinity prediction methods. For binding energy, change in Gibbs free energy ΔΔ(G) score between CLCuMB-βC1 and GhSnRK1 protein was determined. Negative value for ΔΔG (Table 1) indicated interaction between full length proteins as lower free energy change (more negative value) is directly proportional to more stable protein complex.

**Table 1:** Values for change in Gibbs free energy (ΔΔG).

ΔΔG kcal/mol			

Domains	PPA-Pred	PRISM	PRODIGY
KD	-9.2	-14.02	-46.5
**UBA**	-**11.3**	-**19.2**	-**66.3**
**AIS**	-**11.5**	-**23.7**	-**71.8**
CTD	-8.7	-14.8	-49.1


*Bold values for UBA and AIS domain showing higher negative value in terms of rrG which means they are more interacting.*

In case of domain-based interaction, ΔΔG values for KD and CTD were less negative while UBA and AIS (bold) values were more negative shown in Table 1. These high negative values also indicated possible binding of these proteins especially UBA and AIS domains with CLCuMB.

### *In silico* Identification of Protein–Protein Interactions

In addition to the binding affinity prediction, we used multiple binding site prediction methods based on protein sequence and structure. Binding score from sequence-based methods and predicted relative accessible surface area (RASA) values from structure-based methods identified residues mostly from UBA and AIS domains and very few (13 out of 269) residues were predicted from KD domain. This interaction was further studied in detail with docking and machine learning based methods. Docking methods such as ZDOCK 3.0.2 (Pierce et al., 2014) and Docking2 at ROSETTA v3.2 (Lyskov et al., 2013) predicted ten models along with their expected confidence values. To evaluate models from both methods, residues within 5 Å between both chains were selected as possible binding sites. Our analysis revealed a high tendency for UBA and AIS domains to be involved in the interaction using ZDOCK. Moreover, result from Docking2 showed a region in the C-terminal of UBA domain and N-terminal of AIS domain are potentially involved in binding (Supplementary Figures S2A,B). In case of βC1 of CLCuMV, residues from the main α-helix and myristoylation-like motif (101–108) forming a loop-turn structure are predicted to be involved in the interaction. Sequence-based machine learning method, PPiPP (Ahmad and Mizuguchi, 2011), also pointed to the central region of βC1 for binding. In case of SnRK1, most of the residues from KD and UBA domains were predicted in case of binding site with βC1. Structure-based machine learning method, PRISM (Baspinar et al., 2014) extracted data from surface and core of the rigid body structures of both βC1 and SnRK1 gene (Supplementary Figure S2C). Another sequence- and structure-based method, PAIRPred (Minhas et al., 2014) predicted the binding site in AIS domain based on heatmap (Supplementary Figure S2D). For βC1, similar results were obtained as previously from ZDOCK and Docking2 method.

**FIGURE 3:**
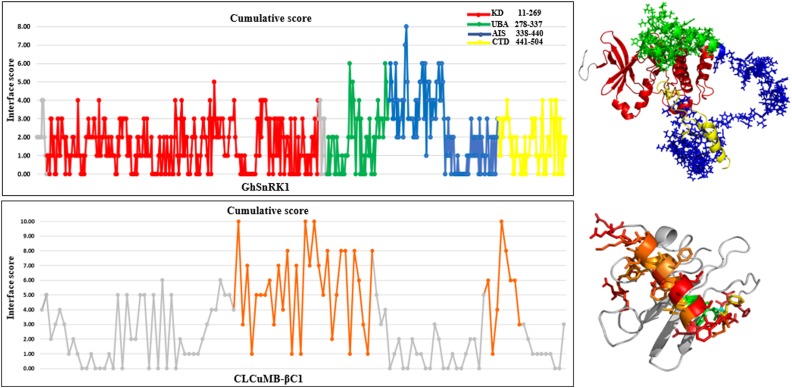
Cumulative score of all prediction methods for CLCuMB-βC1 binding with host protein GhSnRK1. Graphical representation corresponds to the binding region predicted from bioinformatics analysis. Data shows that residues specifically from C-terminal of UBA (green) and N-terminal of AIS (blue) domain possess high score for binding. For virus protein CLCuMB-βC1, all sequence and structure prediction methods identified binding site mainly in α-helix region shown in orange color. Light and dark gray color indicates GhSnRK1 and βC1 protein, respectively. Red color indicates interacting residues in GhSnRK1 and blue color indicates interacting residues in βC1

Predictions obtained with all computational interaction methods were combined to identify potential interacting site(s) in GhSnRK1 protein using majority consensus. This bioinformatics approach was applied to CLCuMB-βC1 with GhSnRK1 and the control, TYLCCNB-βC1 with SiSnRK1 (Supplementary Table S2). For host protein, UBA and AIS domains were predicted to be involved in binding, while in case of the satellite protein, the interaction site in βC1 associated with CLCuMB turned out to be the same as in TYLCCNB (Supplementary Table S3), indicating potential binding site in its α-helix and myristoylation-like motif (Figure 3). It should be pointed out that both betasatellite proteins belonging to two different viruses had only 30% sequence identity as well as low structural similarity to each other. However, predicted regions in βC1 to be involved in α-helix formation, suggesting that the α-helix forms a primary binding pocket with its targeted protein in multiple hosts.

We also used Consurf (Ashkenazy et al., 2010) to study the evolutionary conservation. Multiple sequence alignment identified less conservation score for C-terminal of UBA and N-terminal of AIS domains (Supplementary Table S4). While the KD domain including serine-threonine positions constitute highly conserved regions and deletion of Ser-Thr residues could cause loss of function of GhSnRK1 protein thus limiting the interaction with CLCuMB-βC1. However, SnRK1 causes phosphorylation of TYLCCNB-βC1 mainly through Ser-33 and Thr-78 residues in βC1. While CLCuMB-βC1 possesses positive charged Lys at position 78 instead of a non-charged Thr-78, it remains to be seen what other residues are involved in phosphorylation. Moreover, it was observed that substitution-deletion in UBA and AIS domains resulted in weak interaction between GhSnRK1 and βC1 using interface alanine scanning (Kortemme et al., 2004). In this *in silico* mutagenesis study, chain “A” shows virus protein and “C” shows host protein structure in the complex, and yellow colored residues in pdb# are the amino acids mainly from UBA and AIS domain (Supplementary Table S5). Value of int-id in these residues is zero, indicating unbound amino acids with the interacting partner βC1. Similarly, score in DDG ΔΔ(G) is also positive possessing low binding energy with the residues present in virus protein. This data was further verified using sequence tolerance tool (Smith and Kortemme, 2011). This tool predicted the relative frequencies for GhSnRK1 residues, identified as “tolerated” without compromising the stability of a protein structure. Introducing Alanine (334-AAAAAA-339) shows tolerance frequency in amino acids 336 and 337 was low compared to other four amino acids (Supplementary Figure S3A). However, insertion of Alanine did not cause conformational changes in GhSnRK1 protein structure (Supplementary Figure S3B). Altogether, positive values for DDG complex and stable GhSnRK1 structure after substitution is a better choice for mutagenesis study.

### Identification of Binding Sites Within Domains

**FIGURE 4:**
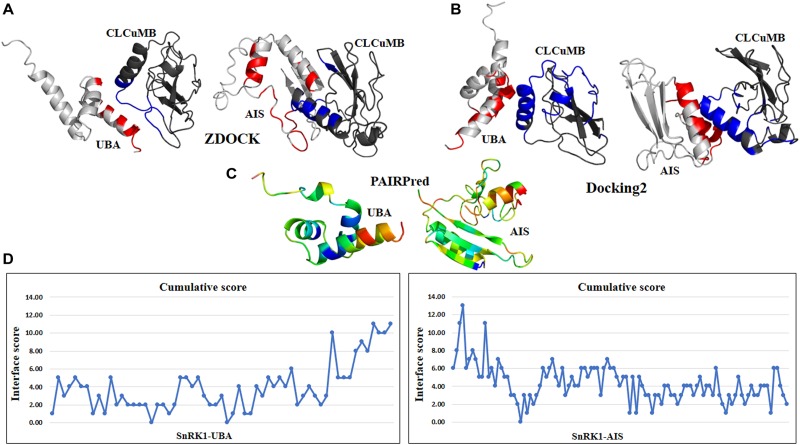
Interaction prediction analysis within a domain using sequence and structure approach. **(A)** ZDOCK predicted C-terminal region of UBA domain in binding with virus protein. In AIS domain, N-terminal region forming a loop structure is predicted for interaction. **(B)** Docking2 at Rosetta dock predicted similar results for UBA domain as previously in ZDOCK. For AIS domain, N-terminal and few residues from C-terminal are found in high binding affinity. **(C)** Using PAIRPred, residues with red hot color in UBA and AIS domain are involved in interaction. For CLCuMB-βC1, residues mainly from α-helix are found in interaction. **(D)** Consensus of all binding and interface methods identified C-terminal of UBA domain and N-terminal of AIS domain for binding with virus protein CLCuMB-βC1.

The above predictions were further verified at the individual domain level. For this propose, all four domains were analyzed individually with CLCuMB-βC1. This analysis has identified that C-terminal of UBA and N-terminal of AIS domains are involved in interaction with CLCuMB-βC1. Machine learning and docking methods including PAIRPred, ZDOCK and Docking2 at ROSETTA showed that UBA and AIS domains have maximum likelihood for interaction (Figure 4A–C), while KD and CTD domains were predicted to have a lower probability for interaction. Therefore, we overlaid the predicted accessible surface area score from all sequence- and structure-based methods for UBA and AIS (Figure 4D) which verified previous results obtained from full length virus-host protein interaction prediction. Deletion of these two domains reduced ΔΔG value for both viral proteins, showing a weaker affinity. Results from this analysis indicated that *in silico* interaction prediction could be useful in predicting binding between two proteins. Moreover, independently of any reference-based analysis, this computational approach is useful in determining potential protein–protein interactions especially at the domain level.

### *In vivo* GhSnRK1 and CLCuMB-βC1 Interaction Using Y2H

**FIGURE 5:**
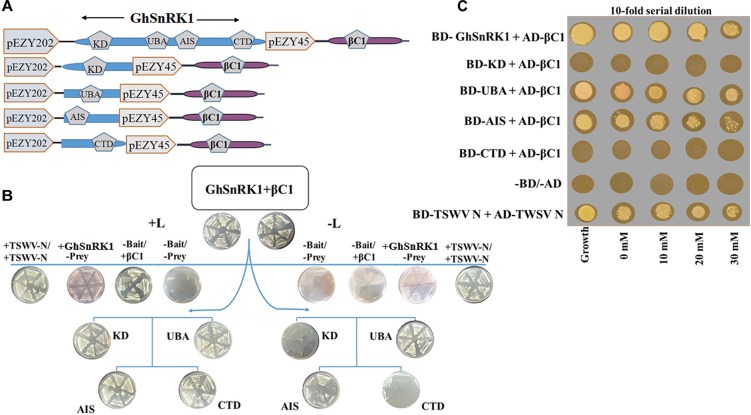
Validation of computer aided results for interaction using yeast two hybrid assay. **(A)** Schematic representation of constructs designed for yeast two hybrid. Full length GhSnRK1 (506 aa), its domains KD (259 aa), UBA (59 aa), AIS (104 aa), and CTD (65 aa) fused with LexA DBD in pEZY202. CLCuMB-βC1 (357 bp) protein is fused with B42 TAD in pJG45. **(B)** First two plates on top panel shows positive result for full length GhSnRK1 and CLCuMB-βC1 on SD-Ura-His-Trp (+L) and SD-Ura-His-Trp-Leu (–L) media. Second lane shows results for autoactivation and positive control (TWSWV-N). Third and forth lane shows domain based results. Only UBA and AIS domain shows positive colonies on both +L and –L media, validating *in silico* results. **(C)** Results were further verified on SD-Ura-His-Trp-Leu media provided with different concentration of 3-amino-1,2,4-triazole (3-AT).

Y2H assay was used to verify the *in silico* interaction predictions between full length and domain-based interactions between GhSnRK1 and CLCuMB-βC1. Basic Gateway cloning strategy is shown in Figure 5A where bait plasmids were transformed, and colonies were obtained on +L medium. Bait-prey transformation on –L media showed strong interaction between full length GhSnRK1 and CLCuMB-βC1 (Figure 5B), thus validating the computational prediction for both proteins. Based on the three-dimensional structure analysis of SnRK1 protein, it was investigated that which domain, within the full length protein, was responsible for interaction with CLCuMB-βC1. Similar results were observed as predicted from the *in silico* analysis. Very weak or almost no interaction was observed between the KD domain and CLCuMB-βC1 (Figure 5B), and the same results were obtained for the CTD domain (Figure 4B). However, UBA and AIS domains showed strong interaction in Y2H assay on appropriate selection media (Figure 5B). Because of the LEU2 reporter gene in EGY48/pSH18-34 strain, autoactivation screening onto SD media lacking Leu showed growth reduction after 2– 3 days (Figure 5B). This screening step confirmed the positive interaction along with all the controls used in the experiment. Adding 3-amino-1,2,4-triazole (3-AT) to yeast media and then increasing the dose of 3-AT resulted in an enhanced growth of positive colonies only. Furthermore, only the UBA and AIS domains expressed strong interaction on SD-His/-Leu/-Trp/-Ura + 3-AT (Figure 5C). These results confirmed that CLCuMB-βC1 interacts with GhSnRK1 through UBA and AIS domains, while CTD and KD domains seem to have limited or no role in the interaction, confirming the predictions in the *in silico* template-based study.

### *In planta* GhSnRK1 and CLCuMB-βC1 Interaction Study

**FIGURE 6:**
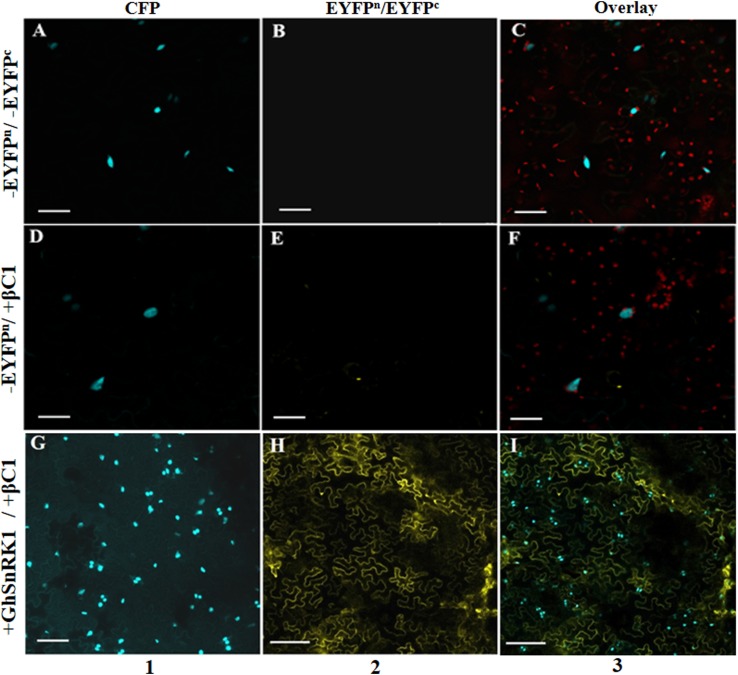
Bimolecular fluorescence complementation assay for co-localization of GhSnRK1 protein with CLCuMB-βC1 in epidermal cells of *Nicotiana benthamiana* leaves. Images were captured at 48 h dpi. **(A–C)** First row represents agroinfiltration of empty vector in *N. benthamiana*. **(D–F)** Second row shows transformation of CLCuMB-βC1 protein in cEYFP with empty nEYFP vector to confirm autofluorescence. **(G–I)** Third row shows positive interaction of GhSnRK1 with CLCuMB-βC1 in nEYFP:cEYFP vector. Maximum projections used x20 with scale bar 50 μm, (1) CFP-H2B, (2) YFP, (3) overlay of both markers.

**FIGURE 7:**
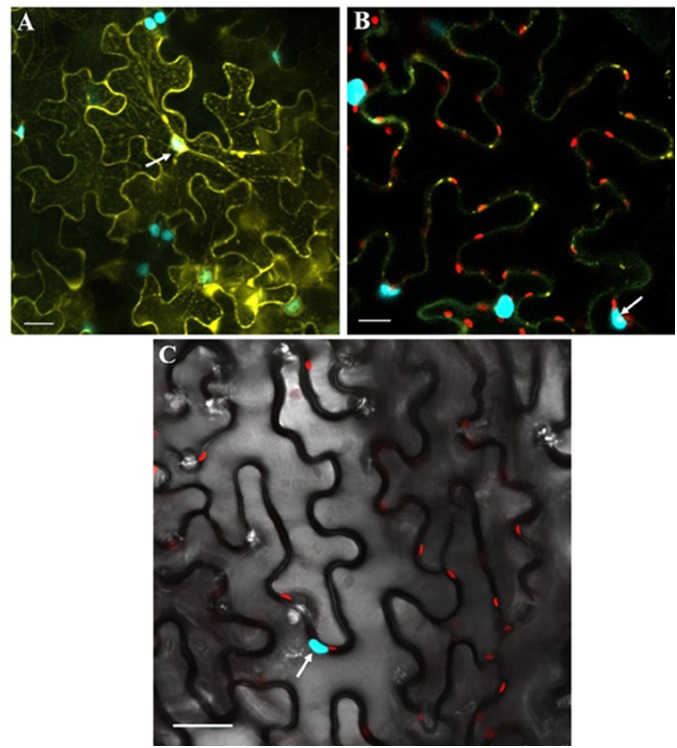
Co-expression of GhSnRK1 and CLCuMB-βC1 at higher magnification during BiFC assay. All images indicate interaction is spread in the cytoplasm and around the nucleus. **(A)** Confocal micrograph showing subcellular localization of GhSnRK1 with CLCuMB-βC1 in nucleus. **(B)** Predominant interaction was determined with clear expression of nucleus and nucleolus marked specifically with CFP-H2B and chlorophyll in red. **(C)** Bright field image of interacting pair in *N. benthamiana* leaves with high resolution. Panels **(A,B)** 20× magnification, 25 μm scale bar. Panel **(C)** 40× magnification, 50 μm scale bar. White arrow indicates nucleus location.

BiFC assay was performed to confirm the results of *in silico* studies for CLCuMB-βC1 protein interaction with GhSnRK1. For this assay, GhSnRK1 and all four domains were separately fused with the n-terminal fragment of pSITE-EYFP-C1. CLCuMB-βC1 was introduced into the C-terminal fragment of pSITE-YFP-C1. All constructs were separately agroinfiltrated into wild type and transgenic *N. benthamiana* plants expressing cyan fluorescent protein carrying nuclear marker histone 2B (CFP-H2B) for subcellular localization of the nucleus/nucleolus in the leaves (Martin et al., 2009). Under confocal microscopy, no fluorescence signal was observed for the control carrying the empty N-terminal and C-terminal fragments of YFP vector (Figure 6A–C). Constructs with the empty N-terminal fragment and CLCuMB-βC1 on C-terminal produced little or no BIFC signal in both CFP and YFP markers (Figure 6D–F). Full length host protein GhSnRK1 showed strong binding affinity with the CLCuMB-βC1 and interaction was localized to cytoplasm and nuclear compartment of wild type leaves as well as CFP-H2B-based marker leaves after 48 h (Figure 6G–I). This sub-cellular localization of these proteins was determined using PredictProtein (Yugandhar and Gromiha, 2014) showing SnRK1 and βC1 in cytosol which means their presence occurs in two organelles including nucleus and cytoplasm. A higher magnification (20×) showed the nuclear location with cytoplasmic veins (Figure 7A,B) and highlights the interaction in cytoplasm and its epithelial cells. Bright-field image of both interacting partners with nucleus surrounded by red chlorophylls confirmed the interaction between GhSnRK1 and CLCuMB-βC1 proteins (Figure 7C).

**FIGURE 8:**
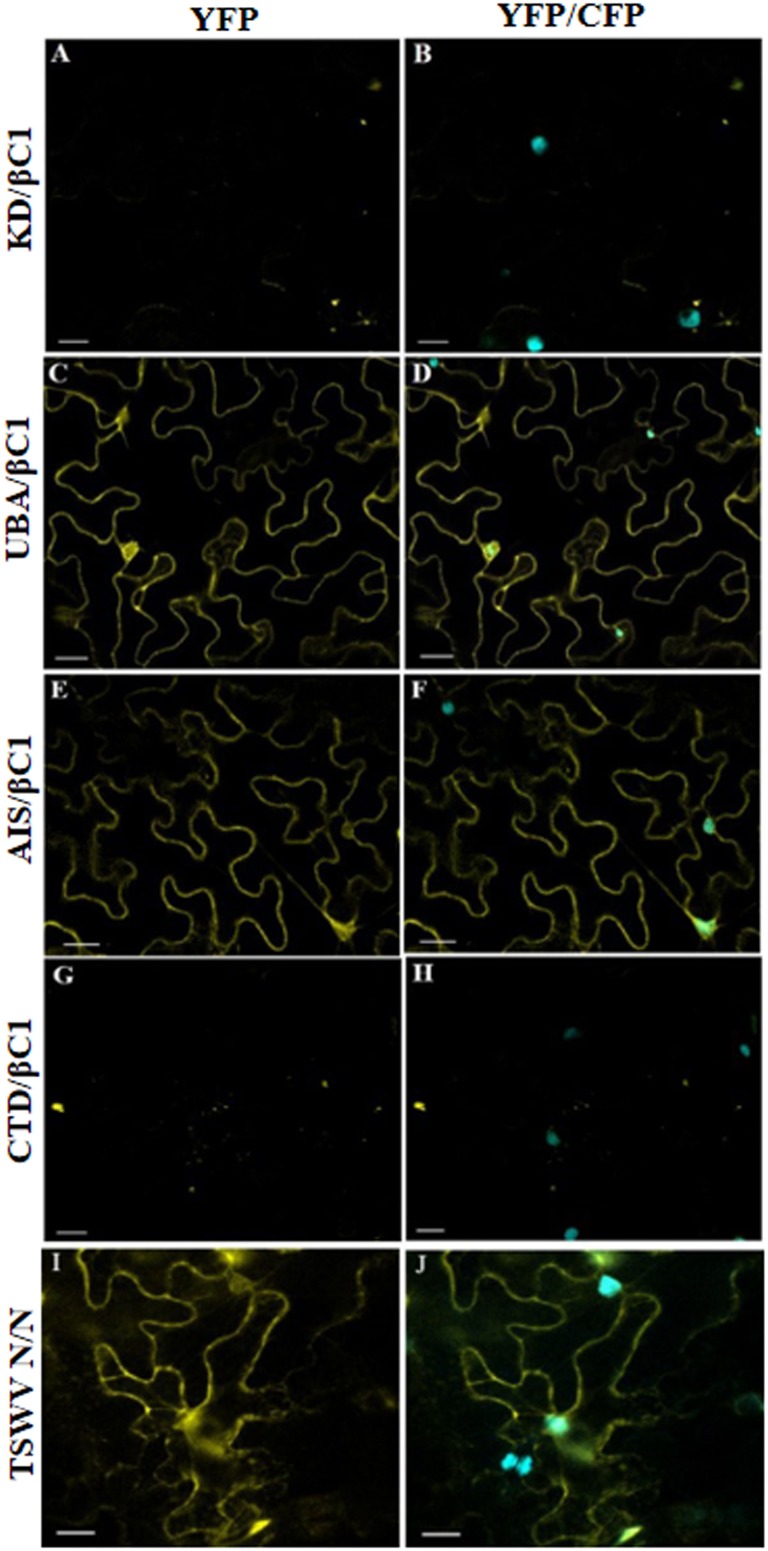
*In vivo* domain-based interaction of GhSnRK1 with CLCuMB-βC1 protein. All the constructs were agroinfiltrated into *Nicotiana benthamiana* plants at an OD_600_ of 0.8, and confocal microscopy study was done after 48 h post infiltration. **(A,B)** KD domain does not show any positive signal for interaction with CLCuMB-βC1 on both YFP and CFP-H2B marker. **(C–F)** UBA and AIS domains produces strong signals for CLCuMB-βC1. **(G,H)** CTD domain shows zero to almost no signals for CLCuMB-βC1 protein. **(I,J)** TSWV N proteins self-interaction confirms the successful transformation event and true signals under confocal microscopy. All the images were acquired at 20× zoom option. Scale bar = 50 μm.

Furthermore, we have investigated domain-based interaction of GhSnRK1 with CLCuMB-βC1 using the BiFC assay. For this purpose, all the domains of GhSnRK1 and CLCuMB-βC1 were independently expressed in wild type and CFP-H2B marker-based *N. benthamiana* plants. No interaction signals were observed for KD with CLCuMB-βC1 (Figure 8A,B), while weak signals were observed for CTD (Figure 8G,H). However, UBA and AIS domains showed strong interactions under confocal microscopy using YFP and CFP markers (Figure 8C–F). This indicated that the residues present in both UBA and AIS domains play a role in interaction between GhSnRK1 and CLCuMB-βC1, while KD and CTD domains do not seem to, which was again in correlation with the results obtained from the bioinformatics analyses and Y2H assay. TSWV N protein’s interaction was used as a positive control to verify the transformation event and post-infiltration experimental steps (Figure 8I,J).

### *In vitro* GhSnRK1 and CLCuMB-βC1 Interaction Using Pull Down Assay

**FIGURE 9:**
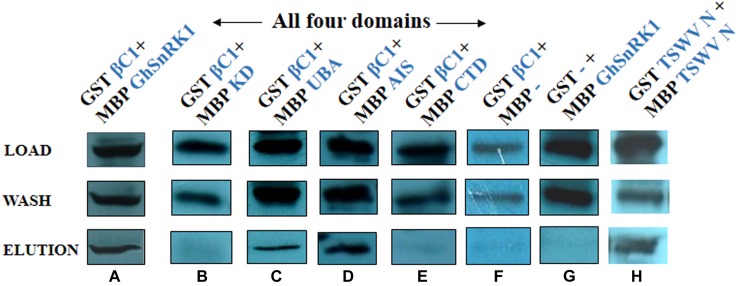
Identification of domain-based interaction between GhSnRK1 and CLCuMB-βC1 using pull down assay. The upper most panel shows protein samples are initially loaded to the column buffer as crude extract, middle panel shows wash samples that are purified with column buffer to remove unbound proteins and lower most panel represents the eluted samples that are purified from amylose resin with maltose. **(A)** The purified sample carries MBP-tagged GhSnRK1 fused with GST-tagged CLCuMB-βC1, **(B)** MBP-tagged KD fused with GST-tagged CLCuMB-βC1, **(C,D)** MBP-tagged UBA and MBP-tagged AIS domain were fused with GST-tagged CLCuMB-βC1, **(E)** MBP-tagged CTD fused with GST-tagged CLCuMB-βC1, **(F,G)** MBP-alone fused with GST-tagged CLCuMB-βC1, and MBP-tagged GhSnRK1 fused with GST-alone was used as negative control, **(H)** MBP-tagged TSWV-N fused with GST-tagged TSWV-N used as positive control. All protein samples were developed on x-ray film in Western blot using anti-GST primary antibody.

Pull down assays were carried out to confirm the observed interaction of CLCuMB-βC1 with GhSnRK1. Purified protein samples were resolved using Western blot. Incubation of the blotted membrane with anti-GST antibody showed the bands for interacting partners after ECL-based detection, indicating that the virus interacts and potentially interferes with the modulating pathway of GhSnRK1 (Figure 9A). Next, we investigated the domain-based binding affinity for CLCuMB-βC1. Weak signals were detected for CLCuMB-βC1 interaction with KD and CTD domains (Figure 9B–E) which further validated our results from Y2H and BiFC assays. Brighter bands in eluted samples for UBA and AIS showed that both domains have strong binding affinity with CLCuMB-βC1 (Figure 9C,D). These results confirmed that CLCuMB-βC1 interacts with GhSnRK1, and UBA and AIS domains are involved in the binding. GST-tagged CLCuMB-βC1 with MBP alone and MBS-tagged GhSnRK1 with GST alone were examined as two sets of negative controls (Figure 9F,G) and TSWV-N protein (Figure 9H) was expressed as a positive control.

## Discussion

Here we have shown, through multiple lines of evidence, that CLCuMB-βC1 serves as a pathogenicity determinant by interacting with GhSnRK1 through UBA and AIS domains. One of the highlights of our study was the demonstration of the relatively high reliability of the various bioinformatics algorithms in first predicting the interacting domains using machine learning and docking methods which were then validated through three independent experimental approaches. Plants infected by Tomato yellow leaf curl China-betasatellite (TYLCCNB) overexpressed SnRK1 protein through UBA and AIS domains resulting in a delay in symptom induction and reduced DNA level by phosphorylating βC1 (Shen et al., 2011). This previous work has been used in a parallel to identify binding sites for SnRK1 gene for another betasatellite protein CLCuMB using computational biology to determine its function in cotton.

It has been studied that SnRK1-α subunit in plants comprises of four domains (KD, UBA, AIS and CTD) that acts as an important key regulator against abiotic stresses especially in abscisic acid signaling (Cutler et al., 2010). Against the stress response, KD supports catalytic mode for SnRK1α protein to retain its structure and function. While UBA, AIS, and CTD domains act as a linker region to interact with other regulatory subunits (β,γ) of SnRK family to maintain energy metabolism (Broeckx et al., 2016). *G. hirsutum* coded SnRK1 protein also consist of four domains with a size of 506 aa, and in order to overcome some of the logistical constraints in analyzing the data, we first adopted a computational approach based on multi-variant approach which helped us to identify domains that are involved in interaction with CLCuMB-βC1. PPI study investigates the interaction among interfacial residues of two proteins using sequence and structure information (Rice et al., 2000; Xiao et al., 2013). Sequence-based approach extracts the information from orthology, gene ontology, and molecular interaction databases to predict the function of an unknown protein using function of an immediate neighbor protein (Ma et al., 2011). Sequence analysis predicted a higher negative value of ΔΔG for GhSnRK1 and CLCuMB-βC1, showing strong binding association between them. *In silico* deletion of UBA and AIS domains in the GhSnRK1 protein reduced the ΔΔG value, predicting a weak interaction among them. Binding site prediction approach uses protein secondary structure, solvent accessibility and conservation score from both sequence and three dimensional structure to identify putative domain based binding sites (Ohue et al., 2013). From binding site methods, it was observed that residues in SiSnRK1 at positions Phe-172, Thr-214, Phe-271, Val-301, Ser-446 have the same binding score for GhSnRK1 at positions Phe-170, Thr-212, Phe-269, Val-299, and Ser-449. However, residues present at 51–65 and 96–106 in TYLCCNB-βC1 and CLCuMB-βC1 have a higher likelihood for interaction.

Sequence-based methods rely on knowledge-based data that requires prediction for each residue present in a protein. Binding site prediction methods alone may not generate reliable information especially where the structure has not been determined experimentally using X-ray crystallography or nuclear magnetic resonance (NMR) spectroscopy (Xiao et al., 2013). Here we have successfully used a multi-pronged approach based on sequence conservation analysis, energetics, binding site and interface prediction methods to first predict the interaction between viral and host proteins, identify domains within the host protein responsible for binding with viral protein and furthermore, localize the residues in the interacting domain that are responsible for their binding affinity. All three computational approaches were applied first to full length host protein and then domain-based independent interactions were studied with viral protein to formulate an overall picture of binding site. In addition, protein docking data showed that amino acids at the C-terminal of UBA (residues at position 333–336) and N-terminal (residues at position 337–343) of the AIS domain are responsible for binding activity, suggesting that these two domains may phosphorylate CLCuMB-βC1 after virus infection. Moreover, it remains to be studied that mutagenesis study including alanine-substitution of these predicted residues either weakens the host protein interaction with viral protein or not.

To validate the predictions obtained by computational analyses, three independent molecular techniques were used to know the interaction between GhSnRK1 and CLCuMB-βC1 at the domain level. Y2H is an *in vivo* tool to investigate possible interacting partners, identifying protein role at a cellular level (Rodríguez-Negrete et al., 2014; Lin and Lai, 2017). Y2H data confirmed that SnRK1 of *G. hirsutum* interacts with CLCuMB-βC1. Further, positive results for UBA and AIS domains on 3-AT-SD/-His/-Leu/-Trp/-Ura media also confirmed the *in silico* predictions for PPI. Same results were obtained *in planta* BiFC assay. Gateway vectors compatible for *in planta* detection of multiple protein interactions using BiFC system is a robust and rapid method to identify subcellular localization of a protein in organelle (Kamigaki et al., 2016). We performed BiFC experiments using full-length as well as domain-based host proteins with CLCuMB-βC1 using Gateway vectors. Expression pattern driven by CFP-H2B and YFP marker indicated strong signals between GhSnRK1 and CLCuMB-βC1. Within this host protein, no interaction was found between CLCuMB-βC1 and KD and CTD domains, while strong interaction was observed for UBA and AIS domain. These findings indicate that CLCuMB-βC1 protein disturbs the signaling pathway of GhSnRK1 against stress mechanism and overcomes the nutrient metabolism of the host protein for its pathogenicity.

Basically, SnRK1 are protein kinases that are involved in various physiological processes which regulates energy metabolism in plants to provide nutrients against biotic and abiotic stress (Halford et al., 2003; Baena-González et al., 2007). The SnRK (SnRK1, SnRK2, SnRK3) protein in plants act as antiviral agent, producing resistance against pathogen by phosphorylation of either pathogen or host protein (Hulsmans et al., 2016; Bai et al., 2017). Therefore, very likely mode of action after GhSnRK1 binding with CLCuMB-βC1 indicates SnRK1 protein phosphorylates βC1 to attenuate viral infection in the cotton. However, this GhSnRK1 interaction with CLCuMB-βC1 was also tested physically using pull down assay based on affinity purification method (Louche et al., 2017). Protein binding experiment using anti-GST antibody in the pull down assay produced strong signals for GhSnRK1 and CLCuMB-βC1 on PVDF membrane after Western blotting. Again, positive signals were detected only for UBA and AIS domain.

In conclusion, our findings provide new insights into begomovirus–cotton interactions at the molecular level and lays foundation for further studies on structure-function relationships. Our findings suggested that bioinformatics approach could predict potential protein binding sites in viral- and host-coded proteins. And the evidence that CLCuMB-βC1 binds with GhSnRK1 through UBA and AIS domains. Our study also demonstrated that substitution mutants in a host protein could be a better choice to produce resistance against viruses, while at the same time keeping the host protein structure and function stable. Our results have shown that computational methods followed in present study could be useful in predicting and validating PPI. We also postulated that GhSnRK1 function becomes irreversible in the presence of any third protein related to the defense mechanism that may unphosphorylate CLCuMB-βC1, that enhances virus replication and movement caused during CLCuD.

## Future Prospects

The comprehensive bioinformatics analyses that was carried out could facilitate further in depth study on the effects of substitution mutations of predicted binding residues on protein structure and its conformation, to gain further insights into the complex biological mechanisms of the cell. The computational approach combined with experimental verification presented here could be used to further understand the interactions between geminivirus-host and their biological significance. Geminiviruses mainly interfere with plant’s signaling pathways and its defense mechanism. The tools and materials developed in this study could facilitate further studies on fine-structure mapping of various motifs in both viral and host proteins and their role in modulating geminivirus replication and spread in cotton and the interacting partners in cotton. Knowledge gained from such studies could be useful in developing novel virus suppression strategies.

## Author Contributions

HK, RM, MH, and MK did the experimental work. F-u-AM and MF did the bioinformatic analyses. DT provide the TSWV nucleoprotein gene construct. HK, IA, HP, and SM wrote the first draft. IA, SM, and HP conceived the idea. All authors read and approved the final manuscript.

## Conflict of Interest Statement

The authors declare that the research was conducted in the absence of any commercial or financial relationships that could be construed as a potential conflict of interest.
