# Supplementary material for: In silico Prediction and Validations of Domains Involved in Gossypium hirsutum SnRK1 Protein Interaction With Cotton Leaf Curl Multan Betasatellite Encoded βC1
Source: Front Plant Sci. 2019 May 28;10:656. doi: 10.3389/fpls.2019.00656 (PMC6546731; doi:10.3389/fpls.2019.00656)
Supplement: Supplementary file 1 [file Data_Sheet_1.docx]

***In Silico* prediction and validations of domains involved in *Gossypium hirsutum* SnRK1 protein interaction with Cotton leaf curl Multan betasatellite encoded βC1**

Hira Kamal^1,2,3^, Fayyaz-ul-Amir Afsar Minhas^2^, Muhammad Farooq^1^, Muhammad Hamza^1^, Roma Mustafa^1^, Muhammad Zuhaib Khan^1^, Shahid Mansoor^1^, Hanu R. Pappu^3^ and Imran Amin^1,*^

^1^ National Institute for Biotechnology and Genetic Engineering Jhang road Faisalabad, Pakistan

^2^ Pakistan Institute of Engineering and Applied Sciences (PIEAS), Nilore, Islamabad. Pakistan

^3^ Department of Plant Pathology, Washington State University, Pullman, United States of America

^*^ **Correspondance**

Imran Amin

Email : imranamin1@yahoo.com

**Figure S1. *In-silico* interaction prediction approach using sequence and structure-based methods.** Sequence based methods determines protein binding residues using relative accessible surface area (RASA) information for each residue. All the methods together reveal amino acids present either on the surface for interaction or buried in the structure, possessing less chances for binding.

***** Light and dark grey color indicates GhSnRK1 and βC1 protein respectively

** Red color indicates interacting residues in GhSnRK1 and blue color indicates interacting residues in βC1

**Figure S2. Interaction prediction from docking and machine learning methods. (A-B)** Docking methods ZDOCK and Docking2 at ROSETTA dock generated top ten models for virus-host complex. Among top ten predicted models, very few residues from KD and CTD domain are identified in binding. Most of the binding region is predicted from UBA and AIS domain. For βC1, residues present mainly in α-helix and myristoylation-like motif (103-108) are predicted in close contact. **(C)** Machine learning method PRISM identified binding site from UBA domain of GhSnRK1 protein. **(D)** another machine learning method PAIRPred predicted residues in AIS domain at its N-terminal. Based on B-factor information, red hot color is for strong binding and moving towards purple-blue shows weak interaction in color scheme.

**Figure S3. Substitution deletion in GhSnRK1 using sequence tolerance method. (A)** Residues present at 334 to 339 (UBA and AIS domain residues) possess tolerance to be substituted. Introducing substitution with Alanine only or residues recommended above the dashed line in predicted frequency table shows less changes in GhSnRK1 protein structure. **(B)** Parent protein GhSnRK1 before substitution at 334-VSSGYL-339 was observed to be the same structure after substitution with Alanine at this position.
