# Supplementary figures and images for: In silico Prediction and Validations of Domains Involved in Gossypium hirsutum SnRK1 Protein Interaction With Cotton Leaf Curl Multan Betasatellite Encoded βC1
Source: Front Plant Sci. 2019 May 28;10:656. doi: 10.3389/fpls.2019.00656 (PMC6546731; doi:10.3389/fpls.2019.00656)

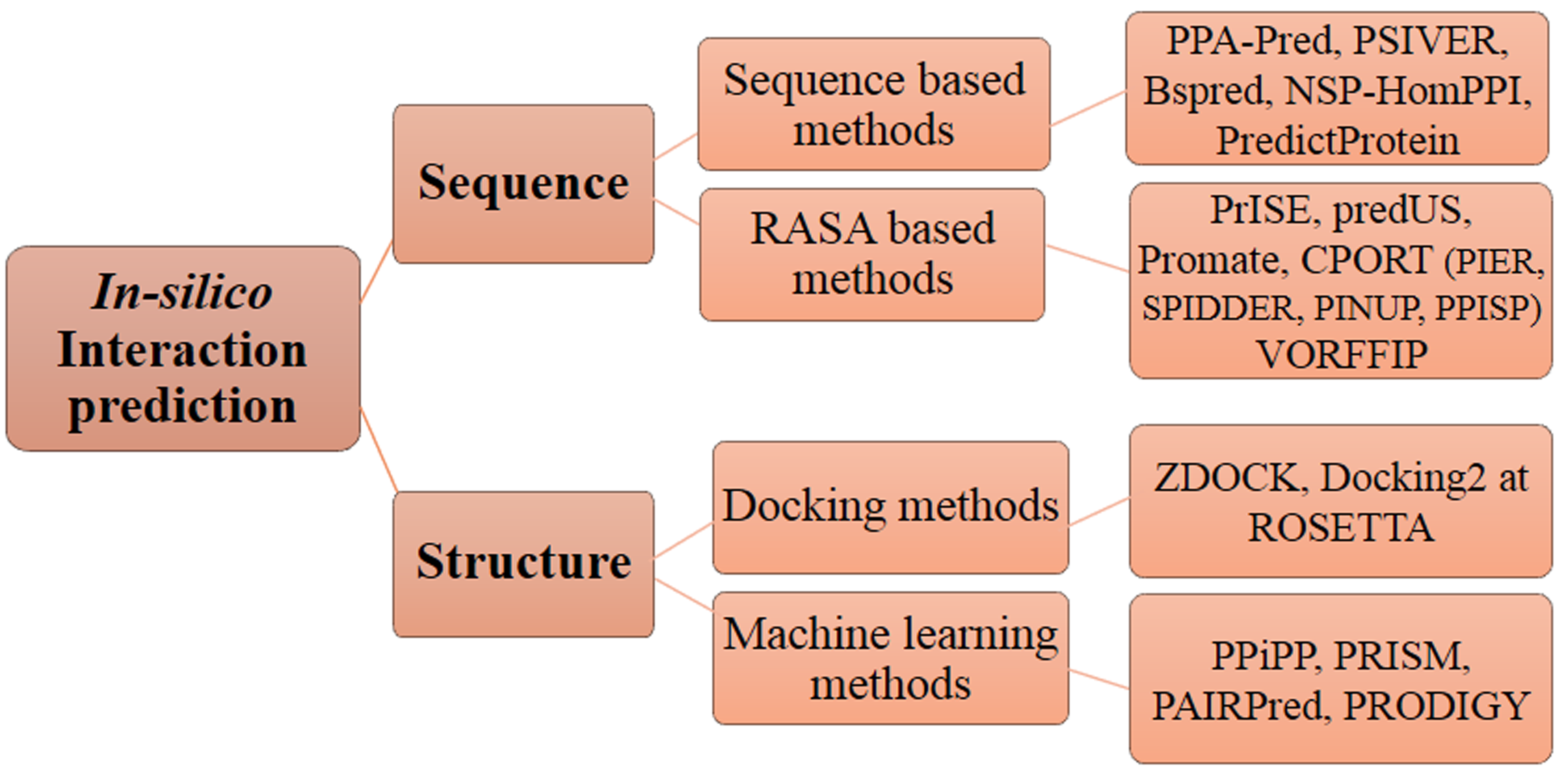

Supplement: Supplementary file 2 [file Image_1.TIF]

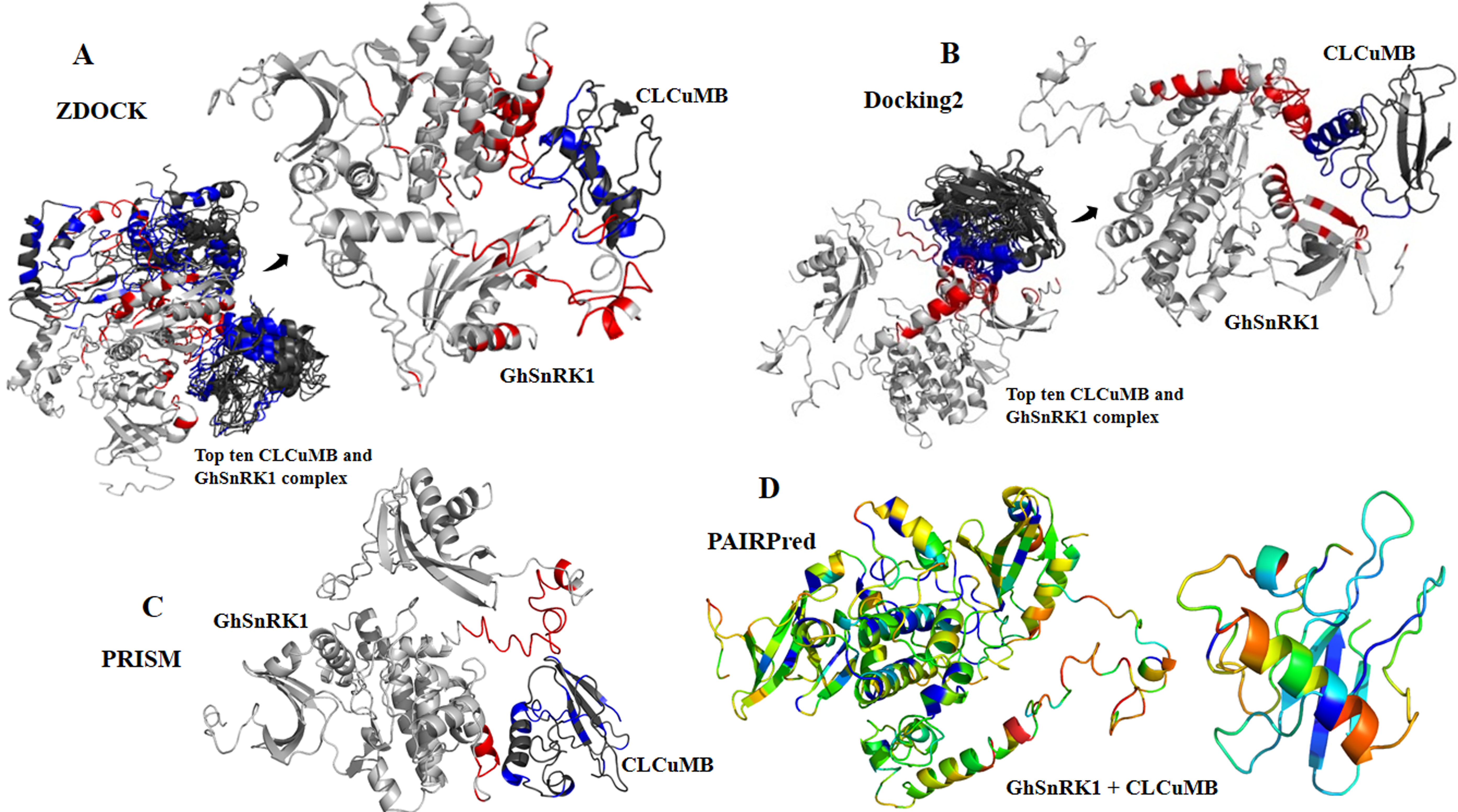

Supplement: Supplementary file 3 [file Image_2.TIF]

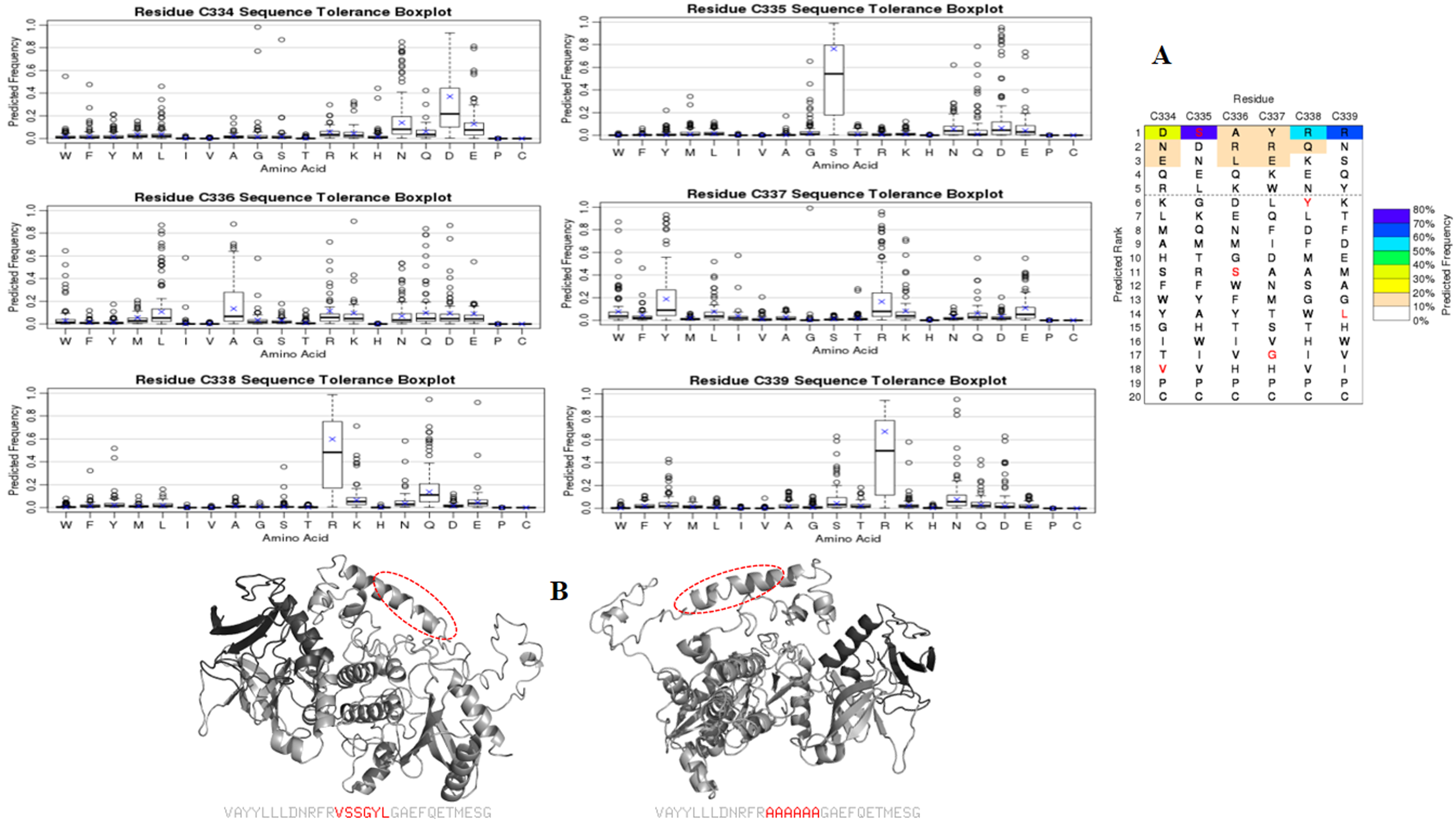

Supplement: Supplementary file 4 [file Image_3.TIF]
